# Supplementary material for: The Complete Mitochondrial Genome of Mytilisepta virgata (Mollusca: Bivalvia), Novel Gene Rearrangements, and the Phylogenetic Relationships of Mytilidae
Source: Genes (Basel). 2023 Apr 13;14(4):910. doi: 10.3390/genes14040910 (PMC10137486; doi:10.3390/genes14040910)
Supplement: Supplementary file 1 [file genes-14-00910-s001.zip › genes-2233695-supplementary.pdf]

**Table S1.** List of species analyzed in this study with their GenBank accession numbers.

| Order | Family | Species | Size (bp) | Accession no. |
|---|---|---|---|---|
| Mytiloida | Mytilidae | *Bathymodiolus aduloides* | 17243 | MT916741 |
| | | *Bathymodiolus azoricus* | 17598 | MT916742 |
| | | *Bathymodiolus brooksi* | 17728 | MT916743 |
| | | *Gigantidas childressi* | 17637 | MT916744 |
| | | *Bathymodiolus japonicus* | 17510 | AP014560 |
| | | *Bathymodiolus sp.* | 18376 | MT916740 |
| | | *Bathymodiolus septemdierum* | 17069 | AP014562 |
| | | *Brachidontes exustus* | 16600 | NC_024882 |
| | | *Crenomytilus grayanus* | 17582 | NC_044128 |
| | | *Gigantidas haimaensis* | 18283 | MT916746 |
| | | *Gigantidas platifrons* | 17653 | AP014561 |
| | | *Gregariella coralliophaga* | 16273 | NC_044129 |
| | | *Modiolus kurilensis* | 16210 | KY242717 |
| | | *Modiolus modiolus* | 15816 | NC_033537 |
| | | *Modiolus nipponicus* | 15638 | NC_044130 |
| | | *Modiolus philippinarum* | 16389 | NC_035422 |
| | | *Modiolus comptus* | 15591 | MN602036 |
| | | *Arcuatula senhousia* | 20612 | NC_014590 |
| | | *Mytella strigata* | 16302 | NC_056377 |
| | | *Mytilus californianus* | 16730 | GQ527172 |
| | | *Mytilus unguiculatus* | 16642 | NC_024733 |
| | | *Mytilus edulis* | 16745 | MF407676 |
| | | *Mytilus galloprovincialis* | 16780 | FJ890849 |
| | | *Mytilus trossulus* | 18628 | HM462080 |
| | | *Mytilus chilensis* | 16765 | NC_030633 |
| | | *Perna canaliculus* | 16004 | NC_054242 |
| | | *Perna perna* | 16100 | MT588202 |
| | | *Perna viridis* | 16014 | NC_018362 |
| | | *Perumytilus purpuratus* | 16986 | MH330333 |
| | | *Semimytilus patagonicus* | 24347 | MT026713 |
| | | *Mytilisepta virgata* | 14713 | ON193524 |
| | | *Mytilisepta keenae* | 15902 | MK721542 |
| | | *Geukensia demissa* | 15838 | MN449487 |
| | | *Xenostrobus securis* | 18145 | NC_028706 |
| | | *Bathymodiolus puteoserpentis* | 20482 | NC_068738 |
| | | *Brachidontes pharaonis* | 20066 | NC_064131 |
| | | *Gigantidas vrijenhoeki* | 17786 | NC_068739 |
| | | *Modiolus modulaides* | 15422 | NC_067876 |
| Ostreida | Pinnidae | *Atrina pectinata* | 16811 | NC_020028 |
| | Ostreidae | *Ostrea lurida* | 16344 | NC_022688 |
| | | *Ostrea denselamellosa* | 16277 | NC_015231 |
| | | *Ostrea edulis* | 16320 | JF274008 |
| | | *Magallana hongkongensis* | 18617 | MZ337404 |
| | | *Magallana gigas* | 18224 | NC_001276 |
| | | *Magallana nippona* | 20030 | NC_015248 |
| | | *Magallana iredalei* | 22446 | NC_013997 |
| | | *Crassostrea sp.* | 22040 | NC_018763 |
| | | *Crassostrea virginica* | 17244 | AY905542 |

| | | | | |
|---|---|---|---|---|
| | | *Magallana belcheri* | 21020 | MH051332 |
| | | *Saccostrea sp.* | 16285 | KU310921 |
| | | *Saccostrea cuccullata* | 16396 | NC_027724 |
| | | *Saccostrea kegaki* | 16260 | NC_030533 |
| | | *Saccostrea echinata* | 16281 | NC_036478 |
| | | *Saccostrea glomerata* | 16282 | NC_036483 |
| | | *Saccostrea mordax* | 16532 | NC_013998 |
| | | *Saccostrea mytiloides* | 16282 | NC_036479 |
| | Margaritidae | *Pinctada imbricata* | 31435 | KX669229 |
| | | *Pinctada maxima* | 16994 | GQ452847 |
| | Pteriidae | *Pteria penguin* | 17344 | KU552127 |
| Pectinida | Pectinidae | *Argopecten purpuratus* | 16270 | NC_027943 |
| | | *Argopecten irradians* | 16211 | NC_009687 |
| | | *Mimachlamys crassicostata* | 17963 | NC_011608 |
| | | *Placopecten magellanicus* | 32115 | NC_007234 |
| | | *Mimachlamys sanguinea* | 17383 | KF214684 |
| | | *Amusium pleuronectes* | 18044 | MT419374 |
| | | *Argopecten ventricosus* | 16079 | KT161261 |
| Arcoida | Arcidae | *Anadara sativa* | 48161 | NC_024927 |
| | | *Anadara kagoshimensis* | 46713 | KF750628 |
| | | *Tegillarca granosa* | 31589 | NC_026081 |
| | | *Anadara vellicata* | 34147 | KP954700 |
| | Cucullaeidae | *Cucullaea labiata* | 25845 | KP091889 |
| Adapedonta | Hiatellidae | *Panopea globosa* | 15469 | NC_025636 |
| | | *Panopea abrupta* | 15381 | NC_033538 |
